# Predicted Heart Age Among Cancer Survivors — United States, 2013–2017

**DOI:** 10.15585/mmwr.mm7001a1

**Published:** 2021-01-08

**Authors:** Lia C. Scott, Quanhe Yang, Nicole F. Dowling, Lisa C. Richardson

**Affiliations:** ^1^Division of Cancer Prevention and Control, National Center for Chronic Disease Prevention and Health Promotion, CDC; ^2^Divsion of Heart Disease and Stroke Prevention, National Center for Chronic Disease Prevention and Health Promotion, CDC.

Approximately 15.5 million cancer survivors were alive in the United States in 2016 with expected growth to 26.1 million by 2040 (*1*). Cancer survivors are living longer because of advances in early detection and treatment, but face psychosocial, cognitive, financial, and physical challenges (*1*,*2*). Physical challenges include cardiovascular complications, partly because cancer and cardiovascular disease (CVD) share some cumulative risk factors including tobacco use, physical inactivity, obesity, poor diet, hypertension, diabetes, and dyslipidemia (*3*). In addition, many cancer treatments damage the heart, and some cancer types increase risk for developing CVD (*4*). The recognition and management of heart disease in cancer survivors has given rise to the discipline of cardio-oncology, which focuses on the cardiovascular health of this population (*5*). CVD risk has been previously estimated using prediction models, and studies suggest that physician-patient communication using predicted heart age rather than predicted 10-year risk has led to a more accurate perception of excess heart age, encouraged actions to adopt a healthy lifestyle, and improved modifiable CVD risk factors (*6*,*7*). Using the nonlaboratory-based Framingham Risk Score (FRS) to estimate 10-year risk for developing CVD, predicted heart age is estimated from the 10-year risk of CVD (predicted by age, sex, diabetes status, smoking status, systolic blood pressure, hypertension treatment status, and body mass index); it is the age of an otherwise healthy person with the same predicted risk, with all other risk factors included in the prediction model at the normal level (systolic blood pressure of 125 mmHg, no hypertension treatment, body mass index of 22.5, nonsmoker, and nondiabetic) (*6*). Using data from the Behavioral Risk Factor Surveillance System (BRFSS), this study estimates predicted heart age, excess heart age (difference between predicted heart age and actual age), and racial/ethnic and sociodemographic disparities in predicted heart age among U.S. adult cancer survivors and noncancer participants aged 30–74 years using previously published methods (*7*). A total of 22,759 men and 46,294 women were cancer survivors with a mean age of 48.7 and 48.3 years, respectively. The predicted heart age and excess heart age among cancer survivors were 57.2 and 8.5 years, respectively, for men and 54.8 and 6.5 years, respectively, for women, and varied by age, race/ethnicity, education and income. The use of predicted heart age by physicians to encourage cancer survivors to improve modifiable risk factors and make heart healthy choices, such as tobacco cessation, regular physical activity, and a healthy diet to maintain a healthy weight, can engage survivors in informed cancer care planning after diagnosis.

Data were drawn from the BRFSS 2013, 2015, and 2017 survey cycles because CVD-specific modules are conducted in odd-numbered years. CDC pooled results for those years to produce stable estimates. The median combined response rate across states for each year was 45.9%, 47.2%, and 45.9%. The eligible sample included nonpregnant adults aged 30–74 years, who had no self-reported history of CVD, including coronary heart disease, myocardial infarction, and stroke. Among 1,362,270 BRFSS participants, the following were excluded: 337,836 participants outside the age range, 3,927 who were pregnant, 103,658 with self-reported CVD, and 70,453 with missing covariates for blood pressure prediction. Among the remaining 846,396 participants for the analysis, 69,053 (8.2%) were cancer survivors. Cancer survivors were defined as having answered “yes” to the question “(Ever told) you had any other type of cancer?” (i.e., excluding skin cancer) by a doctor, nurse, or health professional. Exclusions based on cancer type, which can be found in the cancer survivorship module (https://www.cdc.gov/brfss/questionnaires/pdf-ques/2017_BRFSS_Pub_Ques_508_tagged.pdf), were not made because the survey included that module for only 2017 and did not survey all states.

To account for the complex sampling design, CDC calculated estimates using weights and strata in SAS (version 9.4; SAS Institute) or SAS-callable SUDAAN (version 11.0; RTI International). Systolic blood pressure was calculated using a previously published method (*8*) with National Health and Nutrition Examination Survey 2011–2012, 2013–2014, and 2015–2016 cycles. Predicted heart age was capped with an upper limit of 100 years (*6*).

Age-adjusted weighted means, prevalences, and 95% confidence intervals were calculated for actual age, predicted heart age, and excess heart age. Prevalence of excess heart age of ≥5 years was calculated by age group, race/ethnicity, educational attainment, and annual household income stratified by sex and cancer-survivor status. Adjusted differences in predicted heart age for cancer-survivor versus noncancer status and racial/ethnic differences in predicted heart age for cancer survivors were calculated using multivariate linear regression. A t-test across all age groups was used to ascertain differences within categories, and pairwise t-tests were used for education, income, and race/ethnicity.

A total of 22,759 men and 46,294 women were cancer survivors, with mean ages of 48.7 and 48.3 years, respectively. The predicted heart age and excess heart age among cancer survivors were 57.2 and 8.5 years, respectively, for men and 54.8 and 6.5 years, respectively, for women. The prevalence of excess heart age ≥5 years was 52.4% for men and 43.6% for women. Among cancer survivors, groups with the highest average excess heart age were those in which persons were aged 60–74 years, were non-Hispanic Black, had less than a high school education, and had <$35,000 annual household income (excess heart age range = 11.6–14.9 years for men and 9.8–14.0 years for women). Prevalence of excess heart age ≥5 years was highest for those aged 60–74 years among both men and women; excess heart age ≥5 years was 3.0 percentage points higher for men and 6.5 percentage points higher for women among cancer survivors than among noncancer participants (Table 1).

**TABLE 1:** Age-adjusted and weighted mean actual age, predicted heart age, and excess heart age, and prevalence of excess heart age ≥5 years, by sex, age, race/ethnicity, education level, and annual household income among cancer survivors aged 30–74 years — Behavioral Risk Factor Surveillance System, United States, 2013, 2015, and 2017

Characteristic	No. of persons	Age, yrs (95% CI)			Prevalence of average excess heart age ≥5 yrs % (95% CI)
		Actual age	Predicted heart age	Average excess heart age	
**Men**					
**Cancer status**					
Cancer	22,759	48.7 (48.5–48.8)	57.2 (56.8–57.6)	8.5 (8.2–8.9)	52.4 (50.2–54.6)
No cancer	345,173	47.8 (47.8–47.8)	55.7 (55.7–55.8)	7.9 (7.9–8.0)	49.4 (49.1–49.7)
**Age group, yrs**					
30–39	632	35.0 (34.6–35.4)	37.9 (37.0–38.8)	2.9 (2.2–3.6)	27.4 (22.4–33.2)
40–49	1,289	44.9 (44.6–45.1)	52.5 (51.6–53.4)	7.6 (6.7–8.5)	51.6 (46.8–56.3)
50–59	4,400	55.1 (55.0–55.3)	66.6 (66.0–67.2)	11.5 (10.9–12.0)	66.1 (63.5–68.7)
60–74	16,438	67.2 (67.1–67.3)	82.1 (81.7–82.5)	14.9 (14.6–15.3)	75.3 (74–76.6)
**Race/Ethnicity**					
White, non-Hispanic	19,440	48.7 (48.5–48.8)	56.7 (56.3–57.2)	8.1 (7.7–8.5)	50.2 (47.8–52.7)
Black, non-Hispanic	1,553	48.7 (48.3–49.1)	60.9 (59.6–62.3)	12.3 (11.1–13.4)	70.4 (61.6–77.9)
Hispanic	700	48.6 (48.2–49.0)	56.9 (55.5–58.3)	8.3 (7.0–9.5)	51.2 (44.3–58.1)
Other	1,066	48.7 (48.2–49.2)	58.1 (56.8–59.5)	9.5 (8.1–10.8)	57.6 (49.1–65.7)
**Education**					
Less than HS	1,226	48.7 (47.9–49.4)	61.1 (59.1–63.0)	12.4 (10.9–14.0)	68.5 (58.2–77.2)
HS	5,657	48.6 (48.4–48.9)	58.7 (58–59.4)	10.1 (9.4–10.7)	60.9 (56.5–65.1)
More than HS	15,876	48.7 (48.5–48.9)	55.8 (55.4–56.3)	7.1 (6.7–7.5)	45.7 (43.2–48.3)
**Annual household income ($)**					
<35,000	5,922	48.6 (48.3–48.9)	60.2 (59.3–61.1)	11.6 (10.8–12.3)	65.7 (61.0–70.0)
≥35,000	14,355	48.7 (48.5–48.9)	55.8 (55.4–56.3)	7.1 (6.7–7.5)	46.2 (43.7–48.7)
**Women**					
**Cancer status**					
Cancer	46,294	48.3 (48.2–48.4)	54.8 (54.5–55.0)	6.5 (6.2–6.7)	43.6 (42.3–44.9)
No cancer	430,699	47.9 (47.9–47.9)	53.2 (53.1–53.2)	5.3 (5.2–5.3)	37.1 (36.8–37.4)
**Age group, yrs**					
30–39	2,522	34.5 (34.3–34.7)	36.0 (35.5–36.4)	1.5 (1.1–1.9)	24.0 (21.4–26.7)
40–49	4,726	44.8 (44.7–45.0)	49.4 (48.9–49.9)	4.6 (4.1–5.0)	40.4 (37.4–43.4)
50–59	11,408	54.6 (54.5–54.7)	63.5 (62.9–64.0)	8.9 (8.3–9.4)	51.4 (49.4–53.3)
60–74	27,638	66.8 (66.6–66.9)	80.7 (80.4–81.1)	14 (13.6–14.3)	68.5 (67.3–69.8)
**Race/Ethnicity**					
White, non-Hispanic	39,085	48.4 (48.3–48.4)	54.2 (53.9–54.4)	5.8 (5.6–6.1)	42.2 (40.9–43.5)
Black, non-Hispanic	2,977	48.5 (48.2–48.8)	60.6 (59.7–61.5)	12.1 (11.3–12.9)	62.6 (57.9–67.2)
Hispanic	1,786	48.0 (47.7–48.3)	54.7 (53.8–55.7)	6.8 (5.8–7.7)	39.4 (34.5–44.5)
Other	2,446	48.2 (47.9–48.5)	54.8 (53.5–56.1)	6.6 (5.4–7.8)	44.0 (38.7–49.5)
**Highest education attained**					
Less than HS	2,879	48.1 (47.9–48.4)	59.4 (58.6–60.2)	11.3 (10.5–12)	58.9 (54.6–63.1)
HS	12,163	48.2 (48.0–48.4)	56.2 (55.8–56.7)	8.0 (7.6–8.4)	50.1 (47.6–52.7)
More than HS	31,252	48.4 (48.3–48.5)	53.3 (53.0–53.6)	4.9 (4.6–5.2)	37.9 (36.4–39.3)
**Annual household income ($)**					
<35,000	16,219	48.2 (48.1–48.3)	58.0 (57.6–58.4)	9.8 (9.4–10.2)	56.1 (54.0–58.3)
≥35,000	23,609	48.4 (48.3–48.5)	52.5 (52.2–52.9)	4.1 (3.8–4.4)	34.2 (32.7–35.8)

**Abbreviations:** CI = confidence interval; HS = high school.

Among men, adjusted difference in excess heart age was higher among lower income cancer survivors and non-Hispanic Black cancer survivors than among noncancer participants. Among women, the adjusted difference in excess heart age decreased with age and was higher among lower education, lower income, non-Hispanic Black, and Hispanic cancer survivors than among noncancer participants (Table 2).

**TABLE 2:** Adjusted difference in excess heart age and 95% confidence intervals (CIs) comparing cancer versus noncancer group by sex, age, race/ethnicity, education level, and annual household income for adults aged 30–74 years—Behavioral Risk Factor Surveillance System, United States, 2013, 2015, and 2017

Characteristic	Difference* in excess heart age, yrs (95% CI)	
	Men	Women
**Total^†^**	0.7 (0.4 to 1.0)	0.6 (0.3 to 0.8)
**Age group, yrs** ^§^		
30–39	−0.3 (−1.0 to 0.4)	1.5 (1.1 to 1.9)
40–49	1.6 (0.8 to 2.5)	1.3 (0.8 to 1.8)
50–59	0.8 (0.3 to 1.4)	0.6 (0.1 to 1.2)
60–74	0.6 (0.2 to 0.9)	0.1 (−0.3 to 0.4)
P-value**^¶^**	0.16	<0.001
Highest education attained**^,††^		
Less than HS	1.6 (0.4 to 2.7)	1.4 (0.5 to 2.3)
HS	0.8 (0.3 to 1.3)	0.4 (−0.1 to 0.8)
More than HS	0.5 (0.2 to 0.8)	0.5 (0.2 to 0.8)
**Annual household income ($)^§§^**		
<35,000	1.5 (0.9 to 2)	1.1 (0.7 to 1.5)
≥35,000	0.4 (0.1 to 0.7)	0.3 (−0.1 to 0.6)
P-value^¶¶^	<0.001	0.001
Race/Ethnicity***^,†††^		
White, non-Hispanic	0.4 (0.1 to 0.7)	0.2 (0.0 to 0.4)
Black, non-Hispanic	1.8 (0.9 to 2.7)	2.3 (1.3 to 3.3)
Hispanic	0.9 (−0.5 to 2.2)	1.2 (0.1 to 2.3)
Other, non-Hispanic	2.1 (0.6 to 3.6)	1.9 (0.4 to 3.4)

**Abbreviation:** HS = high school.

* Difference was calculated as excess heart age among noncancer participants subtracted from excess heart age among cancer survivors.

^†^ Adjusted for age, education, and annual household income.

^§^ Adjusted for age, education, and annual household income, with an interaction term of age-by-cancer status to estimate cancer status difference in excess heart age by age group.

^¶^ P-value based on t-tests across the age group.

** Adjusted for age, education, and annual household income, with an interaction term of education-by-cancer status to estimate cancer status difference in excess heart age by education group.

^††^ Based on pairwise t-tests, the following comparisons were significantly different: less than high school versus high school (p = 0.25 for men; p = 0.04 for women); less than high school versus more than high school (p = 0.07 for men; p = 0.06 for women).

^§§^ Adjusted for age, education, and annual household income, with an interaction term of annual household income-by-cancer status to estimate cancer status difference in excess heart age by household income level.

^¶¶^ P-value based on pairwise t-tests.

*** Adjusted for age, education, and annual household income, with an interaction term of race/ethnicity-by-cancer status to estimate cancer status difference in excess heart age by race/ethnicity.

^†††^ Based on pairwise t-tests, statistical significance was established as p<0.05. The p-values for each comparison were as follows: White, non-Hispanic versus Black, non-Hispanic (p = 0 for men; p = <0.001 for women); White, non-Hispanic versus Hispanic (p = 0.53 for men; p = 0.08 for women); White, non-Hispanic versus other (p = 0.03 for men and p = 0.02 for women); Black, non-Hispanic versus Hispanic: (p = 0.24 for men; p = 0.14 for women); Black, non-Hispanic versus other (p = 0.76 for men; p = 0.70 for women); Hispanic versus other: (p = 0.23 for men; p = 0.43 for women).

The difference in predicted heart age between Hispanic and non-Hispanic White cancer survivors was small and not statistically significant for most subgroups, as opposed to a much larger and mostly statistically significant difference between Hispanic and non-Hispanic Black survivors. In addition, disparities were larger among female cancer survivors than among male cancer survivors. The adjusted difference in excess heart age between non-Hispanic Black and non-Hispanic White female cancer survivors increased with age, education level, and income (Table 3). Disparities between non-Hispanic Black and non-Hispanic White persons were greater for women (7.4 years) than for men (4.3 years). The largest differences in excess heart age between non-Hispanic Black and non-Hispanic White women were among those aged 50–59 years (9.2 years) and 60–74 years (8.8 years). Excess heart age differences for each education level were greater for non-Hispanic Black women, with the largest difference occurring for the highest education level (more than high school). Among non-Hispanic Black and non-Hispanic White women with annual household income >$35,000, the difference in excess heart age was 2.4 years higher than that among those with lesser income. Overall, excess heart age is similar among Hispanic and non-Hispanic White cancer survivors, and higher for non-Hispanic Black survivors (Table 3).

**TABLE 3:** Adjusted difference in excess heart age and 95% confidence intervals (CIs) comparing different race/ethnicity groups, by sex, age, education level, and annual household income for cancer survivors aged 30–74 years—Behavioral Risk Factor Surveillance System, United States, 2013, 2015, and 2017.

Characteristic	Difference in excess heart age, yrs (95% CI)					
	Men			Women		
	Black, non-Hispanic versus White, non-Hispanic	Hispanic versus White, non-Hispanic	Black, non-Hispanic versus Hispanic	Black, non-Hispanic versus White, non-Hispanic	Hispanic versus White, non-Hispanic	Black, non-Hispanic versus Hispanic
Total*	4.3 (3.4 to 5.2)	−0.7 (−2.1 to 0.6)	5.0 (3.4 to 6.6)	7.4 (6.5 to 8.4)	−0.7 (−1.8 to 0.4)	8.2 (6.7 to 9.6)
**Age group, yrs^†^**						
30–39	2.5 (−0.3 to 5.2)	−0.7 (−2.9 to 1.5)	3.1 (−0.2 to 6.5)	0.7 (−0.9 to 2.2)	−3.5 (−4.6 to 2.5)	4.2 (2.5 to 5.9)
40–49	2.6 (0.4 to 4.8)	−1.7 (−4.4 to 1)	4.3 (1.1 to 7.5)	4.0 (2.2 to 5.7)	−2.3 (−4.1 to 0.4)	6.2 (3.8 to 8.7)
50–59	3.8 (2.0 to 5.7)	−0.4 (−2.7 to 1.9)	4.3 (1.5 to 7.0)	9.2 (7.0 to 11.5)	−0.1 (−2.7 to 2.4)	9.4 (6.0 to 12.7)
60–74	4.8 (3.6 to 6.0)	−0.7 (−3.0 to 1.6)	5.5 (2.9 to 8.0)	8.8 (7.6 to 10.0)	0.8 (−1.3 to 3.0)	8.0 (5.5 to 10.4)
P-value^§^	0.09	0.81	0.31	<0.001	<0.001	<0.001
Highest education attained^¶,^**						
Less than HS	2.4 (−0.2 to 5.0)	−3.1 (−6.3 to 0.1)	5.5 (1.9 to 9.1)	4.6 (2.1 to 7.1)	−2.6 (−4.8 to 0.3)	7.2 (4.1 to 10.3)
HS	4.2 (2.5 to 5.8)	−1.4 (−3.8 to 1)	5.6 (2.7 to 8.4)	6.3 (4.7 to 7.9)	−1.5 (−3.2 to 0.1)	7.8 (5.6 to 10.0)
More than HS	4.9 (3.6 to 6.1)	1.0 (−0.8 to 2.9)	3.9 (1.7 to 6.1)	8.6 (7.3 to 10.0)	0.8 (−0.8 to 2.4)	7.8 (5.8 to 9.9)
**Annual household income ($)^††^**						
<35, 000	4.1 (2.7 to 5.5)	−0.5 (−2.7 to 1.8)	4.6 (2.1 to 7.1)	6.2 (4.6 to 7.8)	−1.6 (−3 to 0.2)	7.8 (5.8 to 9.8)
≥35,000	4.7 (3.5 to 5.9)	−0.8 (−2.7 to 1.1)	5.5 (3.3 to 7.7)	8.6 (7.3 to 9.9)	0.3 (−1.6 to 2.3)	8.3 (6.0 to 10.5)
P-value	0.55	0.81	0.59	0.02	0.11	0.76

**Abbreviation:** HS = high school.

* Adjusted for age, education, and annual household income.

^†^ Adjusted for age, education, annual household income, with an interaction term of age-by-race/ethnicity to estimate racial difference in excess heart age by age group.

^§^ P-value based on t-tests across the age group.

^¶^ Adjusted for age, education, annual household income, with an interaction term of education-by-race/ethnicity to estimate racial difference in excess heart age by education groups.

** Based on pairwise t-tests, statistical significance was established as p<0.05. The p-values for each comparison were as follows: less than high school versus high school (p = 0.26 for Black, non-Hispanic men versus White, non-Hispanic men; p = 0.40 for Hispanic men versus White, non-Hispanic men; p = 0.98 for Black, non-Hispanic men versus Hispanic men; p = 0.26 for Black, non-Hispanic women versus White, non-Hispanic women; p = 0.46 for Hispanic women versus White, non-Hispanic women; p = 0.74 for Black, non-Hispanic women versus Hispanic women); less than high school versus more than high school (p = 0.09 for Black, non-Hispanic men versus White, non-Hispanic men; p = 0.03 for Hispanic men versus White, non-Hispanic men; p = 0.44 for Black, non-Hispanic men versus Hispanic men; p = 0.01 for Black, non-Hispanic women versus White, non-Hispanic women; p = 0.02 for Hispanic women versus White, non-Hispanic women; p = 0.73 for Black, non-Hispanic women versus Hispanic women).

^††^ Adjusted for age, education, annual household income, with an interaction term of annual household income-by-race/ethnicity to estimate racial difference in excess heart age by household income level.

## Discussion

In this study, both excess heart age and prevalence of excess heart age ≥5 years were higher among cancer survivors than among noncancer participants. Consistent with previous findings, the prevalence of excess heart age was larger among men than among women; however, racial disparities among women were larger than those among men (*7*).

This study also confirms previous findings that few adults meet ideal cardiovascular health metrics, such as not smoking, having normal body mass index, being physically active, eating a healthy diet, and having normal levels of total cholesterol and blood pressure, resulting in excess heart age ≥5 years (*9*). As cancer survivors live longer, more attention can be focused on modifiable risk factors that affect both cancer and CVD (*1*,*2*,*5*). In this study, the prevalence of excess heart age ≥5 years was higher among the following groups: men, cancer survivors with lower income and lower educational attainment, and non-Hispanic Black cancer survivors, particularly women. The findings indicate that wellness plans for all cancer survivors, and particularly the most affected groups, should include a focus on cardiovascular risk.

The findings in this report are subject to at least six limitations. First, BRFSS data are self-reported and thus are subject to recall and social desirability biases (*10*), which might underestimate predicted heart age in all persons. Second, the predicted heart age calculation used model-estimated systolic blood pressure rather than measured systolic blood pressure, which might introduce bias in predicted heart age estimates (*7*). Third, the nonlaboratory-based FRS used in the study might overestimate predicted heart age compared with the laboratory-based FRS (*7*). Fourth, several lifestyle choices such as sodium consumption, physical activity, and diet are not included in the FRS heart age calculation (*7*). Physical activity and diet are specifically linked to both heart disease and cancer, and their exclusion is likely to cause underestimates of predicted heart age in cancer survivors. Fifth, specific risks for some cancer survivors such as cardiotoxic treatment are not considered in the model and are likely to result in further underestimates. Finally, the definition of cancer in this study was nonspecific and could result in misclassification of some study participants.

Adult cancer survivors aged 30–74 years had higher predicted heart age than did noncancer participants, and the degree of excess heart age varied by racial/ethnic and sociodemographic groups. Cancer survivors are living longer and are more likely to experience long-term side effects from therapy that require treatment from multiple types of physicians (*5*). The management of cardiovascular complications and involvement of cardiologists in the integrated approach of cardio-oncology is becoming more important given the steady increase in cancer survival, and the literature suggests the use of a low threshold for referral to a cardio-oncologist or cardiologist (*4*,*5*). The use of predicted heart age by physicians to encourage cancer survivors to improve modifiable risk factors and make heart-healthy choices, such as tobacco cessation, regular physical activity, and a healthy diet to maintain a healthy weight, can engage survivors in informed cancer care planning after diagnosis. In addition, physicians can consider additional components and complications of cancer survivorship, such as mental, social, and financial issues, when formulating a survivorship plan. Cancer survivors might experience numerous macro-level challenges and barriers, which can be included in future analyses to determine potential effect on heart age. By determining and communicating predicted heart age of cancer survivors at a personal level, cancer care teams can provide education to prevent long-term cardiovascular complications and improve quality of life and heart outcomes for cancer survivors.

**Summary**

**What is already known about this topic?**

Cancer and cardiovascular disease (CVD) share long-term risk factors. Physician-patient communication using heart age has been effective in motivating patients to improve modifiable CVD risk factors.

**What is added by this report?**

The predicted heart age and excess heart age among cancer survivors were 57.2 and 8.5 years, respectively, for men and 54.8 and 6.5 years, respectively, for women, and varied by age, race/ethnicity, education, and income. The prevalence of excess heart age ≥5 years was higher among men, cancer survivors with lower income and lower educational attainment, and non-Hispanic Black cancer survivors, particularly women.

**What are the implications for public health practice?**

Health care providers should counsel cancer survivors about ways to reduce modifiable shared risk factors such as tobacco use, physical inactivity, poor diet, hypertension, and obesity that contribute to excess heart age.
